# Fumigant Toxicity of Essential Oils of the Lamiaceae Family Against *Spodoptera frugiperda* Larvae

**DOI:** 10.3390/insects17020162

**Published:** 2026-02-02

**Authors:** Luis Mario Ayala-Guerrero, Francisco Javier Mondragón-Rojas, Anselmo De Jesús Cabrera-Hidalgo, María de los Ángeles Bivian-Hernández, Mayra Beatriz Gómez-Patiño, Petra Andrade-Hoyos, Aarón Mendieta-Moctezuma, Patricia Ibarra-Torres

**Affiliations:** 1 Centro de Investigación en Biotecnología Aplicada, Instituto Politécnico Nacional, Carretera Estatal Santa Inés Tecuexcomac-Tepetitla, Km 1.5, Tepetitla de Lardizábal 90700, Tlaxcala, Mexico; layalag2200@alumno.ipn.mx; 2 Ingeniería en Biotecnología, Universidad Politécnica de Guanajuato, Avenida Universidad Sur 1001, Localidad Juan Alonso, Cortazar 38496, Guanajuato, Mexico; fmondragon@upgto.edu.mx; 3 División en Ingeniería en Innovación Agrícola Sustentable, TecNM-Tlatlauquitepec, Carretera Federal Amozoc-Nautla Km 122+600, Almoloni, Tlatlauquitepec 73907, Puebla, Mexico; anselmo.cabrera@tlatlauitepec.tecnm.mx; 4 SECIHTI-División de Ciencias de la Vida, Universidad de Guanajuato Campus Irapuato-Salamanca, Carretera Irapuato-Silao, Irapuato 36500, Guanajuato, Mexico; mdla.bivianhernandez@ugto.mx; 5 Instituto Politécnico Nacional—CNMN, Unidad Profesional Adolfo López Mateos, Col. Zacatenco, México City 07738, Mexico; mbgomez@ipn.mx; 6 Instituto Nacional de Investigaciones Forestales Agrícolas y Pecuarias (INIFAP), Campo Experimental Zacatepec, Carretera Zacatepec-Galeana s/n, Km 0.5, Colonia IMMS, Zacatepec 62780, Morelos, Mexico; petra.andrade.hoyos@gmail.com

**Keywords:** *Origanum vulgare*, *Lavandula dentata*, *Mentha piperita*, essential oils, *Spodoptera frugiperda*, acetylcholinesterase inhibition

## Abstract

*Spodoptera frugiperda* is a major pest affecting maize production worldwide, and its control relies heavily on synthetic insecticides, which generate environmental risks and resistance. Essential oils (EOs) represent a promising alternative due to their biodegradability and the biological activity of their terpenoid constituents. This study evaluated the chemical composition, larvicidal activity, and acetylcholinesterase (AChE) inhibitory potential of the EOs from *Origanum vulgare*, *Lavandula dentata*, and *Mentha piperita* against *S. frugiperda* larvae at the L1, L2, and L4 instars. Chemical characterization by GC–MS revealed distinct chemotypes: a phenolic/terpenoid profile in *O. vulgare*, a 1,8-cineole-type profile in *L. dentata*, and a menthol-rich composition in *M. piperita*. Larvicidal assays showed dose- and stage-dependent responses, and *M. piperita* EO tended to produce the most robust mortality across instars and times, consistently producing high mortality (75%) at 90 µg·mL^−1^ air concentration. AChE inhibition assays revealed that carvacrol and menthone were the most active constituents with IC_50_ values of 312–330 µg·mL^−1^. Molecular docking supported these results, as carvacrol and menthone showed the most favorable interactions with amino acid residues of the AChE catalytic site. These data support their suitability as candidates for integrated pest management programs targeting *S. frugiperda*. Future research should focus on formulation strategies, synergistic combinations, detoxification mechanisms, and field validation.

## Simple Summary

The fall armyworm (*Spodoptera frugiperda*) is one of the most damaging pests of corn, a staple crop in Mexico and other parts of the world. Its control typically relies on synthetic insecticides, whose continuous use generates environmental risks, resistance, and adverse health effects. A promising alternative is the use of essential oils obtained from aromatic plants, which are biodegradable and contain natural compounds with insecticidal activity. In this study, we evaluated the essential oils of *Origanum vulgare*, *Lavandula dentata*, and *Mentha piperita* on *Spodoptera frugiperda* larvae at three developmental stages. We analyzed their chemical composition, determined their lethal concentrations, and explored a possible mechanism of action through the inhibition of the enzyme Acetylcholinesterase. *M. piperita* essential oil showed the greatest insecticidal activity. Our results demonstrate the potential of these oils for the control of the fall armyworm and suggest their future use in integrated management strategies.

## 1. Introduction

Maize (*Zea mays*) is the second most produced crop worldwide (1.2 gigatons) [1]. Its economic importance lies in the fact that it forms the basis of important staple food products and is cultivated in several countries, such as Mexico. However, in each growing season, maize production is affected by various pests, the main ones being the rootworm (*Diabrotica* spp.), the European and southwestern maize borer (*Ostrinia nubilalis* and *Diatraea grandiosella*), the cutworm (*Agrotis ipsilon*) (primarily the black cutworm), earworms (*Helicoverpa zea*), and subterranean insects [2]. One of the main pests of maize is the cosmopolitan polyphagous insect *Spodoptera frugiperda*, J.E., 1797 (Lepidoptera: Noctuidae), also called the fall armyworm (FAW). The way *S. frugiperda* caterpillars damage corn crops is by chewing the leaves and kernels, resulting in economic losses [3]. In Mexico, this pest can reduce corn production by up to 45%, while in South America, yield losses of up to 72% have been reported [4].

To counteract the damage caused by *S. frugiperda* to corn, farmers use synthetic chemical insecticides. However, their uncontrolled use poses a risk to the environment and the health of users and consumers [5]. To reduce these negative impacts, natural products are an excellent alternative to synthetic pesticides, as they degrade more quickly and leave no residue in food or the ecosystem, making them environmentally friendly [6]. However, it requires optimization through controlled-release formulations and synergistic combinations that improve stability and efficacy. Natural products include essential oils (EOs), extracted from aromatic plants using methods such as hydrodistillation and steam distillation. EO-based bioinsecticides have been successfully evaluated against agricultural pests [7]; their activity is attributed to the high concentrations of key compounds belonging to the classes of terpenes, phenolics, and alkaloids [6]. The main plant families whose EOs have been studied as insecticides against *S. frugiperda* are *Piperaceae*, *Lamiaceae*, and *Verbenaceae*. The most studied plant genus as an insecticide is *Piper* (family *Piperaceae*), followed by *Ocimum* and *Lippia* (family *Lamiaceae*), in the form of EOs against FAW [8].

Recent advances in metabolomic approaches have significantly enhanced the understanding of plant-derived secondary metabolites involved in insect–plant interactions and their potential applications in biological pest control. Untargeted and targeted metabolomics, coupled with chemometric analyses, have enabled the comprehensive characterization of complex phytochemical profiles and the identification of bioactive compounds responsible for insecticidal, antifeedant, or repellent effects. These approaches have been increasingly applied to elucidate the modes of action of EOs and botanical extracts, including disruption of insect nervous systems, interference with detoxification enzymes, oxidative stress induction, and membrane destabilization. Moreover, metabolomic studies facilitate the correlation between specific chemical fingerprints and bioactivity, supporting the rational selection and optimization of plant-based insecticides. Integrating metabolomic insights with traditional bioassays has therefore become a valuable strategy in the development of eco-friendly alternatives to synthetic pesticides for sustainable pest management [9,10].

The insecticidal activity of EOs from *Origanum vulgare* and *Mentha piperita* against *S. frugiperda* has recently been reported [11,12,13,14]. However, this research also includes studies at different developmental stages, the fumigant effect of EOs, and the evaluation of the EO from *Lavandula dentata*. This study evaluated the insecticidal activity of the EOs from *O. vulgare*, *L. dentata*, and *M. piperita* on *S. frugiperda* larvae in the L1, L2, and L4 instars. Activity was verified through chromatographic profiles of the oils, the determination of median lethal concentrations by fumigation, and the evaluation of a possible mechanism of action of the EOs in *S. frugiperda* larvae. The aim of this research is to generate information that will contribute to the development of a natural insecticide product against this pest.

## 2. Materials and Methods

### 2.1. Chemicals and Reagents

The plant species *O. vulgare*, *M. piperita*, and *L. dentata*, sourced from a greenhouse, are certified material. They were cultivated and collected in Ixtacuixtla (19°20′17′′ N, 98°22′26′′ W), Tlaxcala, Mexico, during September 2022. The identification of the plants was carried out by Prof. Laura Garcia. The voucher specimens (9472, 9474, and 9475) were deposited in the TLXM Herbarium of the Center for Research in Biological Sciences of the Autonomous University of Tlaxcala, Tlaxcala, Mexico.

To obtain the EOs, the aerial parts of the three species were segmented into fragments of approximately 10 cm^2^ and extracted by steam distillation in a stainless-steel distillation apparatus (essential distiller, Inoximexico^®^, Guadalajara, Mexico) until no condensation was observed [15]. Subsequently, residual moisture was removed from the EOs with anhydrous sodium sulfate, and the samples were stored at 4 °C in amber glass bottles until later use. EOs were diluted in Tween 80 and water for the bioassays, while for the in vitro acetylcholinesterase inhibition assays absolute ethyl alcohol and water (analytical grade; J.T. Baker, Phillipsburg, NJ, USA) were used.

### 2.2. Chemical Characterization of EOs

Chemical profiles of the EOs of *O. vulgare*, *M. piperita* and *L. dentata* were obtained by GC-MS chromatographic analysis. The EOs were diluted in chloroform at a 1:10 *v*/*v* ratio. Subsequently, 1 µL of the solution was injected into a SCION 456-GC gas chromatograph (GC) (Bruker Daltonics, Billerica, MA, USA) coupled to an EVOQ TQ triple quadrupole mass spectrometer and a CTC PAL-xt autosampler (Bruker Daltonics, Billerica, MA, USA). The mass spectrometer (MS) was set to a range of 50 to 500 mass-charge-1 using MS Workstation software version 8.2.1 (Bruker Daltonics, Inc.). The GC-MS was equipped with a Restek RXI-5 SIL fused silica capillary column, ID 0.32 mm, length 30 m, 0.50 µf (Restek Corporation, Bellefonte, PA, USA). The injector and detector temperatures were 220 °C and 250 °C, respectively. Helium (He) was used as the carrier gas with a flow rate of 1 mL min^−1^. The initial oven temperature was 55 °C for 1 min, then increased to 155 °C at a heating rate of 4 °C min^−1^ and held for 2 min. The temperature was then increased to 255 °C at a heating rate of 20 °C min^−1^ and held for 1 min, for a total analysis time of 34 min per sample. The detected compounds were identified by comparing their retention time and mass spectrum with data from standards included in the NIST Mass Spectral Search Program version 2.2 (National Institute of Standards and Technology, Gaithersburg, MD, USA) (Copyright© 2014, distributed by John Wiley and Sons, Inc., Hoboken, NJ, USA).

### 2.3. Insect Rearing

The *S. frugiperda* colony was obtained from a breeding stock (of specimens collected from corn plots) donated by the Food and Plant Biotechnology Laboratory, University of Guanajuato, Mexico). The rearing process was carried out according to the literature with some modifications [16]. The larvae were maintained on a meridic diet containing 100 mL of distilled water, 1 g of bacteriological agar, 12 g of ground corn, 5 g of brewer’s yeast, 0.5 g of wheat germ, 0.5 g of ascorbic acid, 6.25 g of soybean meal, 0.75 mL of 37% formaldehyde, and 0.075 mg of tetracycline. Adult *S. frugiperda* were fed an aqueous honey solution of 10 g L^−1^. For all bioassays, only caterpillars from the second oviposition of adults maintained in the laboratory were used. The insects were reared and maintained at 25 ± 2 °C, relative humidity of 70 ± 10% and a photophase of 12 h. The *S. frugiperda* larvae were separated into the evaluated instars (L1, L2 and L4) using described morphological characteristics [17].

### 2.4. Fumigant Toxicity Bioassay

Larvicidal bioassays were conducted using a fumigant exposure method in sealed containers [18]. A *S. frugiperda* larva in three developmental stages (L1, L2 and L4) was placed in a closed 30 mL transparent plastic container. Approximately 100 mg of artificial diet was provided in each container to maintain larval viability during the assay. Each experimental unit consisted of 1 larva with 40 replicates per treatment. EOs of *O. vulgare*, *L. dentata*, and *M. piperita*, as well as a positive control insecticide formulation (Chlorpyrifos ethyl (33.8%) + Permethrin (4.8%)), were introduced into the containers without direct contact with the insects, allowing exposure exclusively through the vapor phase. Test concentrations were expressed as µg·mL^−1^ air for EO or insecticide. The final EO concentrations evaluated were 0, 30, 60, 90 and 120 µg EO·mL air^−1^ for all the instars evaluated. A negative control consisting of Tween 80, used as the solvent for both the EOs and the insecticide, was included in all assays. Control treatments were conducted under identical experimental conditions in sealed containers containing diet but without the addition of active compounds.

Larval mortality was recorded after 24, 48, and 72 h of exposure. Mortality data were corrected using Abbott’s formula when necessary to account for natural mortality observed in the negative control. Because insects were exposed to the tested compounds via inhalation of vapors rather than by direct contact or ingestion, toxicity was expressed as lethal concentration values (LC_50_), defined as the concentration in air causing 50% mortality. This metric is appropriate for fumigant bioassays and allows direct comparison between botanical treatments and the synthetic insecticide under identical exposure conditions [18].

### 2.5. Inhibition of Acetylcholinesterase (AChE) of S. frugiperda Extract with EOs

To propose a possible mechanism of action, the in vitro inhibition of AChE of *S. frugiperda* larvae was evaluated using the EOs tested. Approximately 0.1 g of fourth-instar larvae were placed in 1 mL of extraction buffer (0.1 M phosphate buffer, pH 8.0). The mixture was homogenized at 4 °C for 5 min and then centrifuged at 10,000 rpm at 4 °C for 10 min. The supernatant was collected and considered an enzyme extract, stored at 4 °C until use [19].

The insecticide Bendiocarb (2,2-dimethylbenzo-1,3-dioxol-4-yl methylcarbamate), which acts by inhibiting the enzyme acetylcholinesterase, was used as a positive control. EOs and Bendiocarb were dissolved in ethyl alcohol and prepared in five concentration gradients for activity determination. EOs solutions (10 μL) were mixed with the enzyme extract (5 μL) (final EO concentrations of 1, 5, 25, 125, and 500 µg mL^−1^, and insecticide concentrations of 0.1, 0.5, 2.0, 10 and 100 nM) and incubated in a 96-well plate at 37 °C for 2 h. Subsequently, 1.5 mM acetylcholine (ACh) (50 μL) was added, and the mixture was incubated at 37 °C for 5 min. Finally, 0.3 mM 5,5′-dithiobis(2-nitrobenzoic acid) (DTNB) (50 μL) was added to complete the reaction. Residual AChE activity was then measured using a microplate reader at 412 nm.

The percentage inhibition was calculated using Equation 1:

(1) $$Inhibition\;\left(\%\right)=1-(A_{sample}/A_{control})\;\times 100\;$$

where A_sample_ is the absorbance of the sample EOs and A_control_ is the absorbance of the blank [ethyl alcohol in extraction buffer]. EOs IC_50_ values were obtained by linear interpolation/regression using data points bracketing 50% inhibition; SE was derived from the local linear fit [20].

### 2.6. Molecular Docking

Six compounds were selected as ligands, corresponding to the two most abundant constituents of each EO. Ligand-acetylcholinesterase interactions were simulated by molecular docking using AutoDock Vina version 1.2.0. Two-dimensional ligand structures in SDF format were obtained from the PubChem database and converted into three-dimensional structures in MOL2 format using Chem3D version 25.0 after energy minimization. Ligands were prepared with AutoDockTools version 1.5.7 and saved in PDBQT format. Since crystallized structures of AChE from *S. frugiperda* are not available, the AChE structures from *Drosophila melanogaster* (PDB ID: 6XYU) and *Anopheles gambiae* (PDB ID: 5X61) were selected and downloaded from the Protein Data Bank. Protein structures were processed using PyMOL version 2.5.4 and AutoDockTools version 1.5.7 [21,22]. Water molecules, undesired protein chains, and co-crystallized ligands were removed, followed by the addition of hydrogen atoms and Gasteiger charges. The prepared receptor files were finally saved in PDBQT format. The docking search box was defined based on reported active sites and set in terms of coordinates and grid size. Docking simulations were performed at the active sites of the cholinesterases using AutoDock Vina. Docking results were expressed as binding affinity values (kcal/mol), with more negative values indicating stronger predicted binding. Three independent docking runs were carried out for each ligand. Docking poses and ligand–protein interactions were visualized and analyzed using UCSF ChimeraX version 1.10.1 [23].

### 2.7. Data Analysis

Mortality data from fumigant bioassays were corrected for control mortality using Abbott’s formula when necessary. Corrected mortality data were analyzed using Probit regression with a binomial error distribution. For each treatment, larval instar, and exposure time, concentration–mortality relationships were fitted and used to estimate LC_50_ values and their corresponding 95% confidence intervals. When necessary, concentration values were log-transformed to improve linearity of the Probit model. All statistical analyses and graphical representations were performed using R software (version 4.5.1) (R Foundation for Statistical Computing, Vienna, Austria).

Median lethal concentrations (LC_50_) together with their standard errors (SE) and 95% confidence limits (LCL95 and UCL95) were estimated by Probit analysis following Finney [24], for each treatment, larval instar, and exposure time. Model goodness-of-fit was evaluated using the chi-square (χ^2^) statistic, associated degrees of freedom (df), and *p*-values. Differences in toxicity among treatments were inferred based on non-overlapping confidence intervals, and relative power (RP) was calculated by comparing LC_50_ values of EOs with those of the positive control insecticide.

For in vitro acetylcholinesterase (AChE) inhibition assays, IC_50_ values were estimated from concentration–response curves and expressed as mean IC_50_ ± SE. All statistical analyses were performed in R software (R Core Team), using the packages stats for generalized linear modeling, MASS for Probit-based LC_50_ estimation, dplyr for data manipulation, readxl for data import, and ggplot2 for graphical visualization.

## 3. Results

### 3.1. Chemical Analysis of EOs

The yields of *M. piperita*, *O. vulgare* and *L. dentata* EOs were 1.0, 0.82 and 1.20%, respectively. The three essential oils evaluated showed different chemical complexity by GC-MS, where the main constituents identified are presented in Table 1.

The main compounds identified in the *M. piperita* EO were menthol (39.16%), menthyl acetate (22.16%), and *trans*-menthone (18.48%). The major compounds in *O. vulgare* EO were sabinene hydrate (23.67%), carvacrol (7.37%), and β-pinene (5.09%). While for the *L. dentata* EO, approximately 80% of its chemical composition corresponded to the major compounds 1,8-cineole (63.68%), *β*-pinene (13.03%), and α-pinene (4.19%).

### 3.2. Larvicidal Activity of O. vulgare, L. dentata and M. piperita EOs on S. frugiperda

In general, and according to the results obtained from the bioassays, responses were expressed that depended on the concentrations of EOs in the air, the larval stage evaluated, and the time elapsed after EO application.

**Figure 1:**
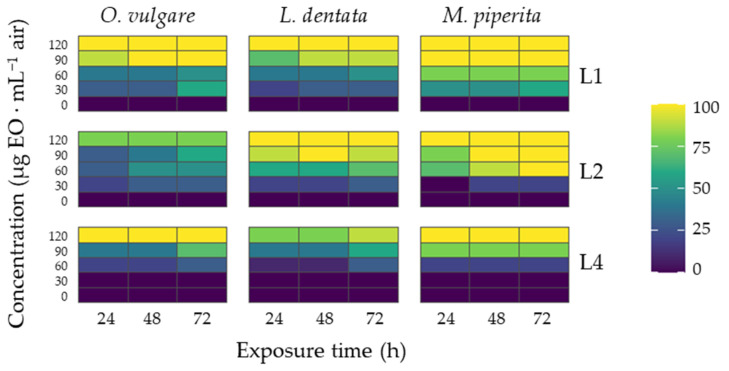
Larval mortality of *S. frugiperda* as a function of EO concentration and exposure time, represented as heatmaps for three instars (L1, L2, and L4) and three EOs (*M. piperita*, *O. vulgare* and *L. dentata*). Each panel shows mortality (%) across the tested concentrations (0.00–120 µg EO·mL^−1^ air) and exposure periods (24–72 h). Increasing color intensity corresponds to higher mortality levels.

The larvicidal effects of *M. piperita*, *O. vulgare* and *L. dentata* EOs against *S. frugiperda* exhibited clear and consistent trends across larval developmental stages (L1, L2, and L4) and exposure durations (24, 48, and 72 h). Heatmap visualization of mortality responses underlines strong concentration–response relationships for the *M. piperita* EO (Figure 1), suggesting that its compounds are biologically active with potential for consideration in integrated pest management (IPM) strategies.

A general trend observed across all EOs was a positive correlation between concentration and larval mortality. At the lowest concentrations, mortality levels were modest, particularly in older instars, but increased progressively with higher doses. At intermediate concentrations, there was a distinct elevation in mortality, and at the highest concentrations tested, mortality approached maximal levels in most instar–time combinations. This pattern is consistent with the dose–response dynamics typically reported in EO bioassays, which show that monoterpene content and oil volatility influence insect toxicity.

Exposure duration exerted a strong influence on mortality outcomes. Across oils and instars, prolonged exposure (72 h) consistently yielded higher mortality compared to 24 and 48 h assessments. Such temporal trends indicate that the active constituents of these EOs may exert cumulative physiological disruption over time, possibly through sustained contact, blockage of the spiracles, or prolonged neurotoxic effects. This aligns with previous findings where extended exposure enhanced efficacy of plant-derived terpenoids against lepidopteran larvae.

Larval susceptibility decreased with increasing developmental age. First instar (L1) larvae exhibited the highest sensitivity, displaying marked mortality even at relatively lower concentrations and shorter exposure times. Second instar (L2) larvae showed intermediate sensitivity, while fourth instar (L4) larvae were generally more tolerant, requiring higher concentrations and longer exposure to achieve similar mortality rates. This trend likely reflects ontogenetic shifts in cuticle thickness, detoxification capacity, and behavioral resilience, consistent with the developmental physiology of *S. frugiperda* and other lepidopteran pests.

Although all three EOs demonstrated larvicidal activity, *M. piperita* tended to produce the most robust mortality across instars and times, followed by *O. vulgare* and then *L. dentata*. *M. piperita* exhibited effective larvicidal activity, albeit with more gradual mortality increases, consistent with reports of menthol and related compounds exerting sublethal and delayed toxic effects. *O. vulgare* may owe its efficacy to high concentrations of carvacrol and thymol, which are known for their strong insecticidal and neuroactive properties. *L. dentata* also delivered high mortality, but the response was somewhat less pronounced, potentially reflecting differences in chemical composition or volatility.

**Figure 2:**
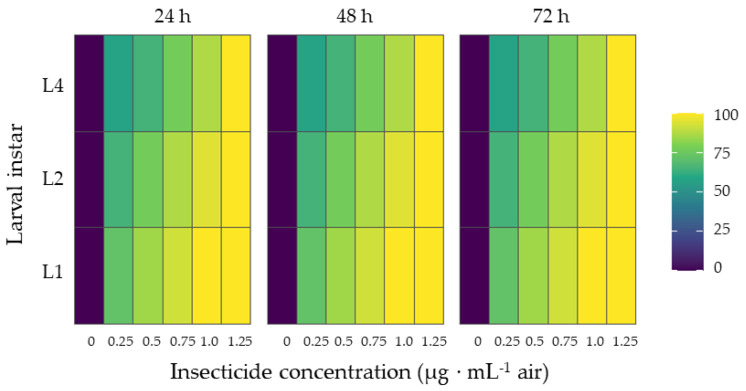
Heatmap representation of the positive control insecticidal effect of a Chlorpyrifos ethyl + Permethrin formulation on *S. frugiperda* larvae. Mean mortality (%) (n = 4 replicates) is shown for three larval instars (L1, L2, and L4) across increasing air concentrations (0–1.25 µg·mL^−1^ air) and exposure times (24, 48, and 72 h). Color intensity reflects the magnitude of larval mortality, illustrating the strong dose- and time-dependent toxicity of the reference insecticide.

The heatmap corresponding to the positive control (Figure 2) shows a strong insecticidal effect of the Chlorpyrifos ethyl + Permethrin formulation against *S. frugiperda* larvae. Mortality increased consistently with both insecticide concentration (0–1.25 µg·mL^−1^ air) and exposure time (24–72 h) across all larval instars (L1, L2, and L4).

First instar (L1) larvae exhibited the highest mortality at all exposure times, reaching near-complete mortality at the highest concentrations after 72 h. Second instar (L2) larvae showed a similar but slightly reduced response, whereas fourth instar (L4) larvae required higher concentrations and longer exposure to achieve comparable mortality levels. In comparison, while the insecticide produced consistently high mortality at all concentrations and time points, the botanical treatments showed greater variability between oils and life stages (Figure 1). Likewise, the evaluated insecticide also showed clear larvicidal effects dependent on concentration and time.

At 72 h and at the highest tested concentrations, some EOs achieved mortality levels approaching those observed for the insecticide, particularly in L1 and L2 larvae. Overall, Figure 2 provides a reference for maximal insecticidal efficacy, allowing the larvicidal activity of the EOs (Figure 1) to be directly contextualized.

### 3.3. Fumigant Toxicity and LC_50_ of EOs on S. frugiperda Larvae

Probit analysis revealed clear fumigant toxicity of the EOs against *S. frugiperda* larvae, with LC_50_ values varying according to larval instar and exposure time. In first instar (L1) larvae, LC_50_ values at 24 h ranged from 30.6 µg EO·mL^−1^ air for *M. piperita* to 53.8 µg·EO·mL^−1^ air for *L. dentata*, with intermediate values observed for *O. vulgare* (47.9 µg EO·mL^−1^ air). Similar trends were observed at 48 and 72 h, with a general decrease in LC_50_ values over time, indicating increased toxicity with prolonged exposure (Table 2). Across all EOs, early larval instars were more susceptible than later instars. In L2 and L4 larvae, LC_50_ values were consistently higher than those observed for L1. The *M. piperita* EO showed the highest relative power value in the L1 stage.

The Cartucho^®^ insecticide (Chlorpyrifos ethyl (33.8%) + Permethrin (4.8%)) exhibited substantially higher fumigant toxicity than the botanical treatments, where LC_50_ values for the insecticide ranged from 0.159 to 0.190 µg mL^−1^ air in the larval instars, with a mortality rate of 100% from 24 h of application (Table S1).

Despite their lower relative potency, the EOs displayed consistent concentration–response and time-dependent toxicity patterns, supporting their potential as fumigant botanical agents and providing a quantitative basis for comparison with the synthetic positive control. Although LC_50_ values were estimated by Probit analysis, goodness-of-fit tests indicated a lack of fit in several cases, and therefore these estimates should be interpreted with caution, particularly when comparing treatments and larval instars. These results establish the insecticide formulation as a highly potent fumigant reference, providing a benchmark for comparison with the botanical treatments.

### 3.4. In Vitro Inhibition of S. frugiperda AChE

In vitro acetylcholinesterase (AChE) inhibition assays using crude extracts from fourth-instar *S. frugiperda* larvae showed concentration-dependent inhibition for all tested samples (Table 3). Among the EOs, *O. vulgare* exhibited the strongest inhibitory activity (IC_50_ = 54 µg·mL^−1^), followed by *L. dentata* (IC_50_ = 144 µg·mL^−1^) and *M. piperita* (IC_50_ = 308 µg·mL^−1^). Among the terpenoids tested, the major bicyclic monoterpene sabinene hydrate, in *O. vulgare* EO displayed substantially lower inhibitory potency (IC_50_ = 2786 µg·mL^−1^), whereas carvacrol showed moderate inhibition (IC_50_ = 312 µg·mL^−1^). A similar trend was observed in the terpenes 1,8-cineole and β-pinene in *L. dentata* EO, as well as for menthone and menthol for *M. piperita* EO, which exhibited a moderate to weak inhibitory effect on AChE.

### 3.5. Molecular Docking Study

Molecular docking analysis revealed that all evaluated terpenes exhibited moderate binding affinities toward AChE from both *D. melanogaster* and *A. gambiae*, with binding energies ranging from −6.67 to −7.60 and −6.25 to −6.67 kcal/mol, respectively (Table 4). Among the natural compounds, menthone, carvacrol, and sabinene hydrate showed the strongest predicted interactions with *D. melanogaster* AChE, whereas slightly lower affinities were observed for *A. gambiae* AChE. As expected, the commercial carbamate insecticide bendiocarb displayed the highest binding affinity against both enzymes, supporting its use as a positive control.

**Figure 3:**
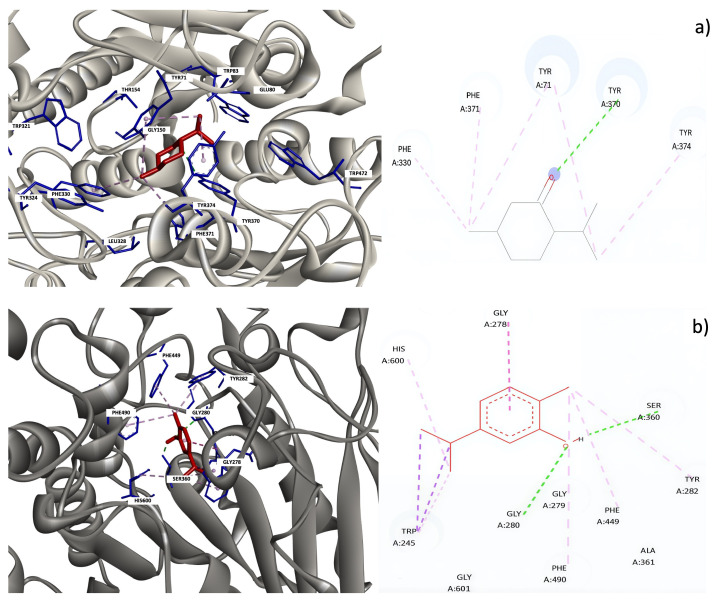
Predicted binding modes and key interactions of menthone (**a**) within AChE from *D. melanogaster* and carvacrol (**b**) within AChE from *A. gambiae*.

Figure 3 shows the predicted binding modes and molecular interactions of selected terpenes within AChE from two insect species. The upper panels correspond to the docking pose of menthone within the active site of *D. melanogaster* AChE, where the ligand is located inside the catalytic pocket and stabilized mainly by hydrophobic and π–π interactions with aromatic residues such as Tyr and Phe, along with additional van der Waals contacts. The lower panels depict the binding mode of carvacrol in *A. gambiae* AChE, showing a similar accommodation within the active site and the formation of hydrophobic interactions, as well as hydrogen bonding interactions with residues including Ser and Gly. These interaction profiles are consistent with the binding affinities obtained from molecular docking and support the potential inhibitory activity of menthone and carvacrol against insect AChE.

## 4. Discussion

Fumigant bioassays revealed that the EOs of *M. piperita*, *O. vulgare* and *L. dentata* were effective against *S. frugiperda* larvae at the L1, L2, and L4 instars, consistently producing high mortality (>75%) at 50–120 µg·mL^−1^ air concentrations. The GC-MS analyses showed that *M. piperita* EO was dominated by monocyclic monoterpenes constituents (menthone, menthol and methyl acetate), a composition that mirrors many contemporary analyses of commercial *Mentha* oils. The preponderance of monocyclic monoterpenes has been related to AChE inhibition, neurotoxic effects in insects and fumigant/contact bioactivity, which explains why these monoterpene-rich *M. piperita* EOs show strong biochemical and insecticidal responses in both in vitro and in vivo assays [25,26,27,28].

*O. vulgare* EO has as its main constituent sabinene hydrate (23%) and a minor content of carvacrol (7%). This is consistent with reports on the sabinene hydrate chemotype of *O.vulgare* EO [29,30]. Terpineol, carvacrol, and thymol are strongly associated with membrane-disrupting and antimicrobial/insecticidal effects; therefore, this chemotype of *O. vulgare* EO could exert its biological activity through the synergistic effect of these monoterpenes [31].

By contrast, the *L. dentata* EO showed a cineole-type profile: 1,8-cineole accounted for ~64% of the oil, with β-pinene (~13%) and γ-terpinene (~3.4%). Cineole-dominant *Lavandula* chemotypes have been associated with high volatility and pronounced fumigant or repellent activity rather than strong contact toxicity mediated by phenolic compounds [32,33,34]. Thus, the biological effect of this *Lavandula* EO is likely to be influenced by rapid vapor-phase activity that favors fumigation and sensory disruption in insects [34,35].

The results in this study indicate that *M. piperita* EO showed the best larvicidal effect against three larval instars of *S. frugiperda*. Using the leaf immersion method, it has been determined that the *C. citratus* EO had greater toxicity than the *M. piperita* EO on third stage larvae of *S. frugiperda*, showing a mortality of 50% with 725.2 mg·L^−1^ and 1024.2 mg·L^−1^ respectively after 48 h of exposure [13]. Likewise, extracts of *M. spicata* of high and medium polarity caused 100% mortality in third-stage larvae [36]. However, in a study where *Mentha* sp. EO was applied orally to first-stage caterpillars of *S. frugiperda*, it showed the lowest toxicity compared to the other treatments evaluated, observing a mortality rate of 50% with a concentration of 0.33 μL·cm^−2^ of diet [37]. The effect of *M. piperita* EO against third-instar larvae of *S. frugiperda* was recently evaluated. The authors reported that, at a 5% concentration, the oil caused 93.3% larval mortality after 72 h of application. Furthermore, at the same concentration, the EO exhibited 80.4% antifeedant activity, suggesting its use for controlling *S. frugiperda* [14].

Regarding the results of the fumigant activity in this study, it has been reported a similar trend of *O. vulgare* EO on *S. frugiperda* applied topically to second-instar larvae at a concentration of 90 μg·larva^−1^ [11]. The monoterpenes carvacrol and γ-terpinene from *O. vulgare* have been reported to mediate the larval feeding preferences of *Spodoptera littoralis*, another species of lepidopteran (moth). The authors reported that the insect reacted to these monoterpenes by modulating the activities of its antioxidant enzymes and gene expression; however, they reported that this was not sufficient to maintain the toxicity of these *O. vulgare* monoterpenes [12]. In addition, carvacrol is well known for its insecticidal effect against *S. frugiperda* larvae by interacting with a variety of insecticidal targets and regulating the food digestion process [11]. Regarding other insects, similar results have been reported when evaluating the contact toxicity of *O. vulgare* EO on *Tribolium confusum* adult larvae, with mortalities between 30 and 60% using a concentration of 0.04 to 0.08 μL·insect^−1^ at 72 h of application [38]. Likewise, a 50% mortality rate of *Tenebrio molitor* larvae has been determined by applying *O. vulgare* EO topically at 3.039 μg·insect^−1^ [39].

On the other hand, the results observed for *L. dentata* EO displayed a dose-dependent and larval stage-dependent pattern. In L1 instar, concentrations of 120 µg EO·mL^−1^ air eliminate survival; in L2, the same concentration produces > 80% mortality. This pattern—greater susceptibility in early stages and the need for higher doses in later instars—is consistent with recently published findings on the control of lepidopteran pests with EOs and botanical extracts, where neonates and early instars are typically more sensitive due to lower body mass, a less developed cuticle, and reduced enzymatic detoxification capacity (P450, esterase), factors that decrease the effective dose per unit of tissue in larger larvae [40]. The chemotype of *L. dentata* could explain the results and dynamics observed in the bioassays. Similar cineole profiles were associated with high percentages of 1,8-cineole with fumigant/repellent activity. Studies on *L. dentata* EO support the notion that the high proportion of eucalyptol (1,8-cineole) results in a highly volatile action profile suitable for fumigation endpoints [34]. In fumigation or vapor phase exposure trials, cineolated EOs show efficacy at relatively low concentrations due to the high volatility of 1,8-cineole. Studies on the formulation and application of EOs for *L. dentata* and closely related species recommend evaluating the exposure method to optimize efficacy according to the objective (fumigation vs. feeding) [41].

The present study demonstrates that the *M. piperita*, *O. vulgare* and *L. dentata* EOs exhibit measurable fumigant toxicity against *S. frugiperda* larvae at different instars, with lethal effects dependent on larval instar and exposure time. Probit analysis revealed clear concentration–response relationships, and LC_50_ values decreased with increasing exposure time, particularly in early instars. Previous research has shown that EOs can exhibit toxic effects against *S. frugiperda*, supporting their potential role in pest management. Larval susceptibility varied with developmental stage, with L1 larvae showing lower LC_50_ values than L2 and L4 larvae. This pattern of early larval stages being more vulnerable to botanical plant-derived toxins has been observed in other EO studies against this pest [42]. Although the EOs evaluated differed in composition, their fumigant toxicity showed comparable trends across instars and times. EO mixtures and constituents have been shown to disrupt insect physiology, supporting the use of EOs as biopesticides [43]. In contrast, while the commercial insecticide exhibited markedly higher fumigant toxicity, with LC_50_ values several orders of magnitude lower than those of the EOs. Recent work highlights the ongoing challenge of insecticide resistance in *S. frugiperda*, underscoring the need for alternative control strategies [44]. Despite being less potent than the synthetic insecticide, EOs showed greater toxicity with prolonged exposure, where at 72 h the treatments approached mortality levels like insecticide in early instars. This aligns with the concept that EOs, while slower acting, can contribute to integrated pest management frameworks. Overall, the results highlight a clear contrast between the high potency of the synthetic insecticide and the moderate fumigant activity of the EOs, reinforcing the relevance of botanical compounds as complementary tools for managing *S. frugiperda* [36].

The in vitro AChE inhibition assays provide mechanistic support for a neurotoxic component contributing to the insecticidal activity of the evaluated EOs against *S. frugiperda*. Among the tested oils, *O. vulgare* showed the strongest AChE inhibitory activity, whereas *L. dentata* and *M. piperita* exhibited moderate to weak inhibition, respectively. This pattern is consistent with previous reports indicating that EOs rich in aromatic monoterpenes display stronger cholinesterase inhibitory activity than oils dominated by non-phenolic monoterpenes [45,46]. Comparison between whole oils and their major constituents highlights the complexity of EO bioactivity. In *O. vulgare*, carvacrol showed good AChE inhibition, while sabinene hydrate exhibited weak activity, suggesting that the inhibitory effect of the whole oil cannot be attributed to a single major compound. Similar data have been reported for *Origanum* chemotypes, where whole oils often show equal or greater bioactivity than their individual constituents due to additive or synergistic interactions [46,47]. For *L. dentata*, the major constituents 1,8-cineole and β-pinene are reported as weak to moderate AChE inhibitors, indicating that the enzymatic inhibition observed for the whole oil likely results from combined effects of multiple constituents rather than dominance of a single terpene [48]. Likewise, the moderate AChE inhibition observed for *M. piperita* oil is consistent with reports showing that menthol and menthone interact moderately with cholinesterases and may exert insecticidal effects through alternative physiological targets [49]. Overall, the AChE inhibition data suggest that interference with cholinergic signaling may contribute to the insecticidal activity of the tested EOs, particularly in the case of *O. vulgare* and *M. piperita,* where their components (carvacrol and menthone, respectively) displayed good inhibitory effect against AChE. However, the moderate IC_50_ values and the limited inhibitory activity of individual terpenes indicate that AChE inhibition alone is unlikely to fully explain the fumigant toxicity observed in vivo. These findings support a multi-target mode of action for EOs, in agreement with recent studies evaluating botanical insecticides against *S. frugiperda* [3].

Although AChE inhibition was evaluated in the present study as an important neurotoxic target, the insecticidal activity of EOs should not be attributed exclusively to this mechanism. EOs are complex mixtures known to act through multiple and complementary pathways. Previous studies have shown that aromatic monoterpenes such as carvacrol and thymol can disrupt insect cell membranes, increasing permeability and causing loss of cellular homeostasis. Additionally, EOs may induce oxidative stress by promoting the generation of reactive oxygen species and impairing antioxidant defenses. Interference with neuronal ion channels and neurotransmitter receptors, including voltage-gated sodium channels, has also been reported for several monoterpenes [18,50,51].

Molecular docking studies provided structural insights into the potential interaction of selected terpenes with insect AChE. All evaluated compounds exhibited moderate binding affinities toward AChE from *D. melanogaster* and *A. gambiae*, suggesting a feasible interaction within the catalytic gorge of the enzyme. The predicted binding modes revealed that menthone and carvacrol were mainly stabilized by hydrophobic interactions and van der Waals contacts with aromatic residues lining the active site, such as Tyr, Phe, and Trp, which are known to play a key role in ligand recognition in AChE [52,53]. In addition, hydrogen bonding interactions with residues including Ser and Gly were observed, potentially contributing to ligand stabilization. Although the binding affinities of the terpenes were lower than that of the reference insecticide bendiocarb, the interaction patterns suggest that these natural compounds may interfere with AChE activity, supporting their proposed role as bioactive constituents contributing to the insecticidal effects reported for essential oils. These findings highlight the relevance of AChE as a potential molecular target for plant-derived terpenes and support their consideration in the development of alternative insect control strategies.

## 5. Conclusions

The essential oils exhibited distinct chemical profiles that influenced their biological responses, where the sabinene hydrate chemotype of *O. vulgare*, the cineolate chemotype of *L. dentata*, and the predominance of oxygenated monoterpenes in *M. piperita* explained the observed variations in larvicidal activity depending on stage and dose. *M. piperita*, *O. vulgare* and *L. dentata* EOs showed consistent fumigant larvicidal activity against *S. frugiperda*, with higher susceptibility in early instars and increased mortality over time. AChE inhibition, particularly by *O. vulgare* EO, suggests a contributory neurotoxic component within a broader multi-target mode of action. Furthermore, the strong inhibition of AChE by carvacrol and menthone confirms its neurotoxic relevance, although in vivo inhibition could still be evaluated in the future. Taken together, these findings support the potential of *M. piperita* and *O. vulgare* EOs as candidates for integrated management strategies of *S. frugiperda*. Its practical use could be optimized through controlled-release formulations and synergistic combinations that improve stability and efficacy. Future studies should address the fumigant activity of major compounds on larvae, enzyme kinetics, detoxification mechanisms, sublethal effects, and field trials to validate their performance and safety under real-world conditions.

## Figures and Tables

**Table 1:** Chemical composition of *M. piperita*, *O. vulgare* and *L. dentata* EOs.

RI ^1^	RI ^2^	Component	Class	Relative Abundance (%)		
				*O. vulgare*	*L. dentata*	*M. piperita*
918	926	β-Thujene	BM	3.65	-	-
923	931	α-Pinene	BM	3.87	4.19	0.35
936	937	Camphene	BM	-	0.22	-
965	964	β-Pinene	BM	5.09	13.03	-
983	986	1-Octen-3-ol	AH	4.11	-	-
987	988	α-Myrcene	AM	4.33	0.55	-
1010	1016	3-Carene	BM	1.85	0.14	-
1021	1026	p-Cymene	ArM	4.06	0.22	-
1026	1028	Limonene	MM	1.35	2.94	2.20
1029	1029	1,8-Cineole	BM	-	63.68	1.04
1039	1044	β-Ocimene	AM	2.06	-	-
1058	1063	*γ*-Terpinene	MM	5.04	-	-
1065	1064	α-Terpinolene	MM	3.02	-	-
1097	1067	Sabinene hydrate	BM	23.67	-	-
1101	1105	Linalool	AM	-	2.62	-
1157	1156	*trans*-Menthone	MM	-	-	18.48
1163	1166	Menthofuran	ArM	-	-	3.32
1174	1172	Terpinen-4-ol	MM	3.75	0.28	-
1186	1185	Menthol	MM	-	-	39.16
1188	1190	α-Terpineol	MM	1.73	1.95	-
1222	1225	Thymol methyl ether	ArM	4.53	-	-
1223	1230	Pulegene	MM	-	-	2.46
1229	1241	Piperitone	MM	-	-	2.12
1252	1274	Mentyl acetate	MM	-	-	22.16
1292	1298	Terpinen-4-yl acetate	MM	3.87	-	-
1298	1302	Thymol	ArM	2.06	-	-
1304	1317	Carvacrol	ArM	7.37	-	-
1409	1415	β-Caryophyllene	BS	2.68	0.21	-
1441	1444	β-cubebene	BS	1.83	-	-
1449	1445	Elemene	MS	2.43	-	-
Total Aliphatic hydrocarbons (AH)				4.11	-	-
Total Aliphatic monoterpenes (AM)				6.39	3.17	-
Total Monocyclic monoterpenes (MM)				18.76	5.17	86.58
Total Bicyclic monoterpenes (BM)				38.13	81.26	1.39
Total Aromatic monoterpenes (ArM)				18.02	0.22	3.32
Total Monocyclic sesquiterpenes (MS)				2.43	-	-
Total Bicyclic sesquiterpenes (BS)				4.51	0.21	-
Total compounds				92.35	90.03	91.29

^1^ Retention indices (RI) were calculated using a series of n-alkanes (C_8_–C_20_) as reference in Elite 5-MS capillary column; ^2^ RI values were compared with those reported in the literature and mass spectra with those available in the NIST 02 database.

**Table 2:** Lethal concentration (LC_50_) values of EOs vapors and a positive control insecticide against *S. frugiperda* larvae across larval instars and exposure times.

Essential Oil	Instar	Time (h)	LC_50_ (µg EO·mL^−1^ air)	SE	LCL95	UCL95	χ^2^	df	*p*	RP	RP LCL95	RP UCL95
*L. dentata*	L1	24	53.8	1.9	50.3	57.6	40.12	14	<0.001	0.0030	0.0032	0.0028
		48	46.1	1.7	42.9	49.6	69.45	14	<0.001	0.0034	0.0037	0.0032
		72	44.2	1.7	41.0	47.5	74.42	14	<0.001	0.0036	0.0039	0.0033
	L2	24	56.2	1.8	52.7	60.0	13.84	14	0.461	0.0034	0.0036	0.0032
		48	43.5	1.6	40.5	46.8	25.28	14	0.032	0.0044	0.0047	0.0041
		72	42.0	1.6	39.0	45.3	21.84	14	0.082	0.0045	0.0049	0.0042
	L4	24	95.2	2.2	91.0	99.6	3.69	14	0.997	0.0026	0.0027	0.0025
		48	95.2	2.2	91.0	99.6	3.69	14	0.997	0.0026	0.0027	0.0025
		72	77.7	1.8	74.3	81.3	37.44	14	<0.001	0.0032	0.0033	0.0030
*M. piperita*	L1	24	30.6	1.8	27.2	34.3	43.61	14	<0.001	0.0052	0.0058	0.0046
		48	29.1	1.9	25.7	33.0	39.35	14	<0.001	0.0055	0.0062	0.0048
		72	25.5	2.1	21.7	30.0	45.81	14	<0.001	0.0062	0.0073	0.0053
	L2	24	61.0	1.6	57.9	64.2	53.49	14	<0.001	0.0031	0.0033	0.0030
		48	38.5	1.3	36.0	41.2	21.18	14	0.097	0.0049	0.0053	0.0046
		72	36.0	1.0	34.2	38.0	13.55	14	0.483	0.0053	0.0056	0.0050
	L4	24	74.8	1.4	72.2	77.5	12.59	14	0.559	0.0033	0.0034	0.0032
		48	74.8	1.4	72.2	77.5	12.59	14	0.559	0.0033	0.0034	0.0032
		72	69.9	1.3	67.4	72.6	8.99	14	0.832	0.0035	0.0037	0.0034
*O. vulgare*	L1	24	47.9	1.9	44.4	51.8	50.59	14	<0.001	0.0033	0.0036	0.0031
		48	44.3	1.6	41.3	47.6	90.99	14	<0.001	0.0036	0.0039	0.0033
		72	34.2	1.9	30.6	38.1	85.27	14	<0.001	0.0047	0.0052	0.0042
	L2	24	97.9	5.1	88.4	108.4	46.63	14	<0.001	0.0019	0.0021	0.0018
		48	66.6	4.2	58.9	75.3	25.40	14	0.031	0.0029	0.0032	0.0025
		72	61.9	3.4	55.5	69.0	31.47	14	0.005	0.0031	0.0034	0.0028
	L4	24	80.0	1.8	76.6	83.7	45.71	14	<0.001	0.0031	0.0032	0.0030
		48	80.0	1.8	76.6	83.7	45.71	14	<0.001	0.0031	0.0032	0.0030
		72	70.3	1.4	67.5	73.1	11.92	14	0.613	0.0035	0.0037	0.0034

Note: LC_50_ values were estimated by Probit analysis. SE = standard error; LCL95 and UCL95 = lower and upper 95% confidence limits; χ^2^ = chi-square goodness-of-fit statistic; df = degrees of freedom; *p* = associated probability value; RP = Relative power (LC_50_ insecticide/LC_50_ EO).

**Table 3:** *In vitro* inhibition of EOs on the crude AChE extract from *S. frugiperda*.

EO/Terpene/Insecticide	IC_50_ ± SE (µg·mL^−1^)
*O. vulgare*	54 ± 0.23
Sabinene hydrate	2786 ± 11.98
Carvacrol	312 ± 1.34
*L. dentata*	144 ± 0.62
1,8-Cineol	737 ± 3.17
β-pinene	7960 ± 34.23
*M. piperita*	308 ± 1.32
Menthol	6140 ± 26.40
Menthone	330 ± 1.42
Bendiocarb	81.25 ± 3.66 (nM)

SE indicates the standard error of the IC_50_ estimate.

**Table 4:** Predicted binding affinities (kcal/mol) of selected terpenes against acetylcholinesterase (AChE) from *D. melanogaster* and *A. gambiae*, obtained through molecular docking analysis.

Compound	PubChem ID	Binding Affinities (kcal/mol)	
		*D. melanogaster* AChE	*A. gambiae* AChE
1,8-Cineol	2758	−6.67	−6.39
β-Pinene	14896	−7.19	−6.35
Menthol	1254	−7.27	−6.59
Menthone	26447	−7.60	−6.61
Sabinene hydrate	22226117	−7.40	−6.25
Carvacrol	10364	−7.47	−6.67
Bendiocarb	2314	−8.45	−7.75

## Data Availability

The original contributions presented in this study are included in the article/Supplementary Materials. Further inquiries can be directed to the corresponding authors.

## Supplementary Materials

The following supporting information can be downloaded at https://www.mdpi.com/article/10.3390/insects17020162/s1, Figure S1: Chromatogram obtained by GC-MS of essential oils extracted by steam drag from fresh *O. vulgare* leaves; Figure S2: Chromatogram obtained by GC-MS of essential oils extracted by steam drag from the leaves and flowers of *L. dentata*; Figure S3: Chromatogram obtained by GC-MS of essential oils extracted by steam drag from fresh *M. piperita* leaves; Table S1: Lethal concentration (LC_50_) values of positive control insecticide against *S. frugiperda* larvae, estimated by Probit analysis larval instars and exposure times.
