# Supplementary material for: Fumigant Toxicity of Essential Oils of the Lamiaceae Family Against Spodoptera frugiperda Larvae
Source: Insects. 2026 Feb 2;17(2):162. doi: 10.3390/insects17020162 (PMC12940607; doi:10.3390/insects17020162)
Supplement: Supplementary file 1 [file insects-17-00162-s001.zip › insects-4104241-supplementary.pdf]

## Supplementary material

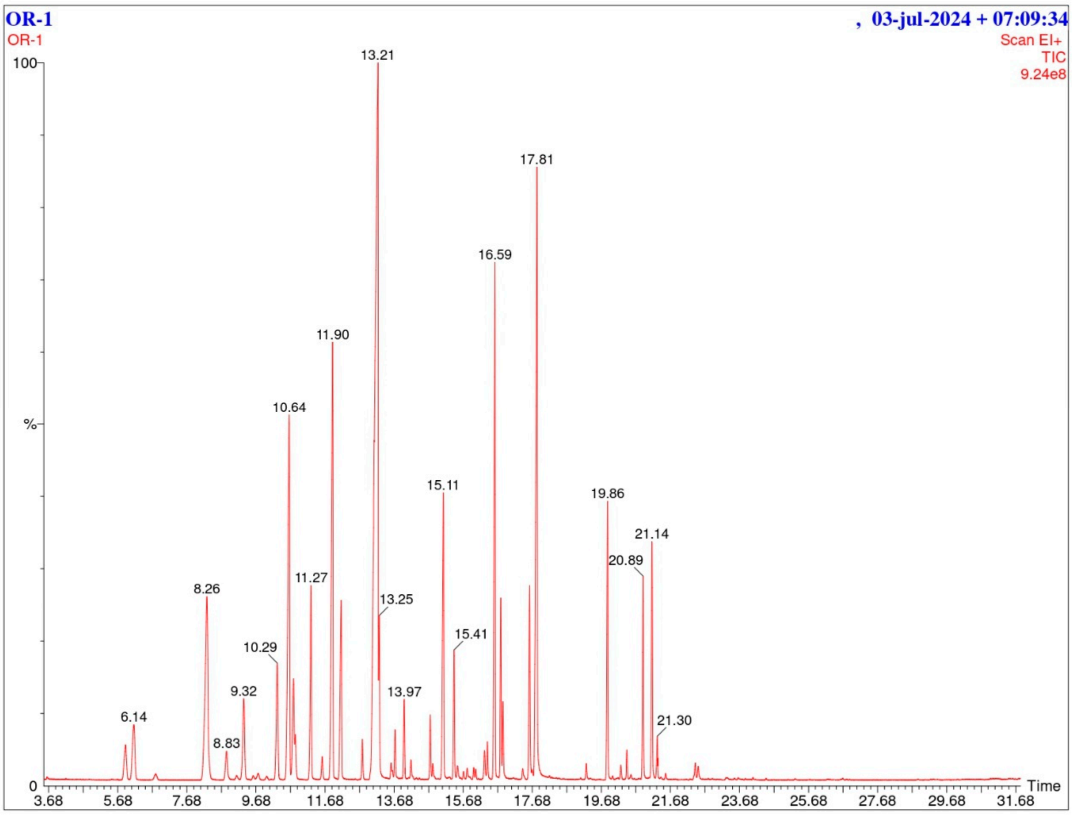


**Figure S1**. Chromatogram obtained by GC-MS of essential oils extracted by steam drag from fresh *O. vulgare* leaves.

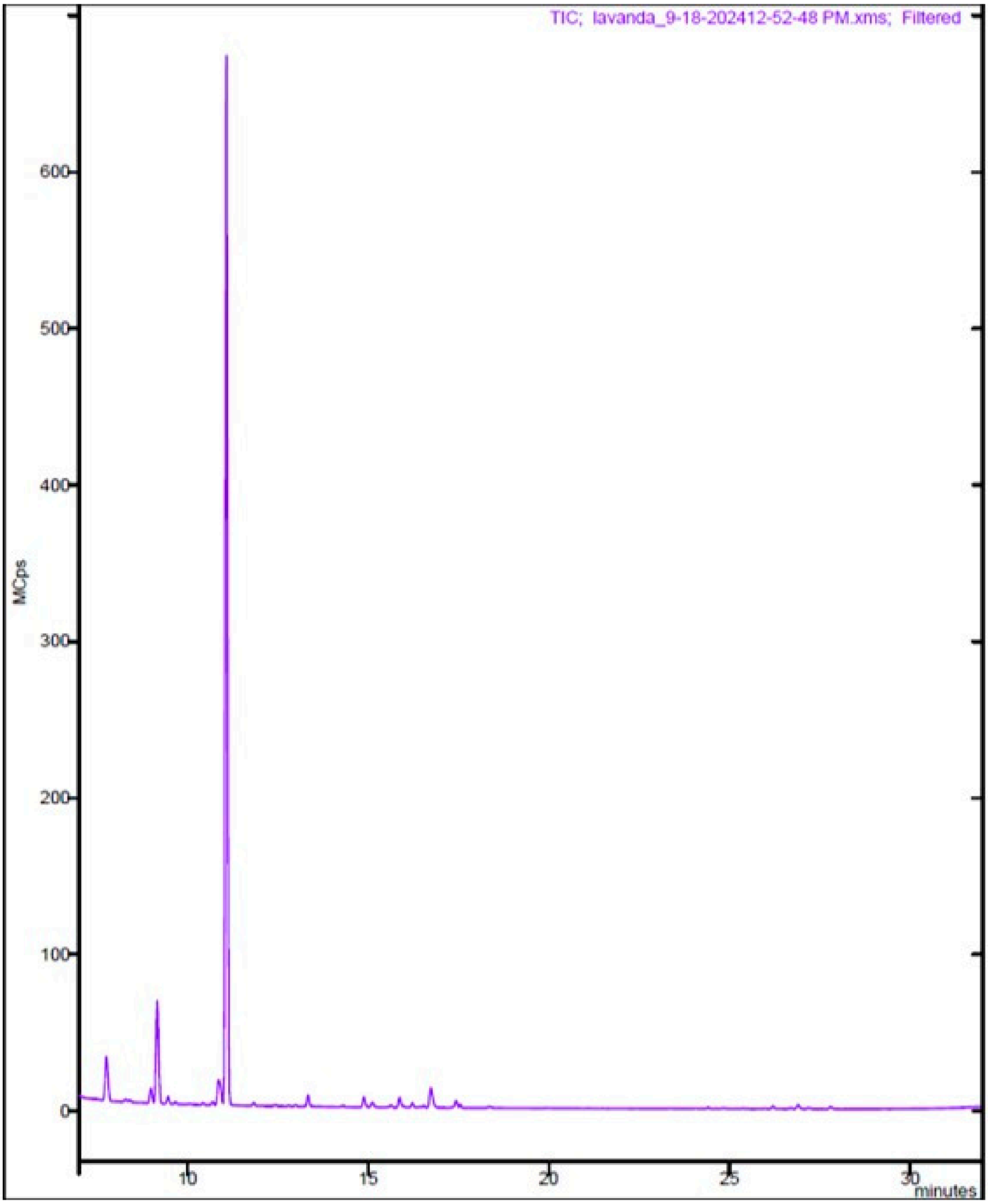


**Figure S2**. Chromatogram obtained by GC-MS of essential oils extracted by steam drag from the leaves and flowers of *L. dentata*.

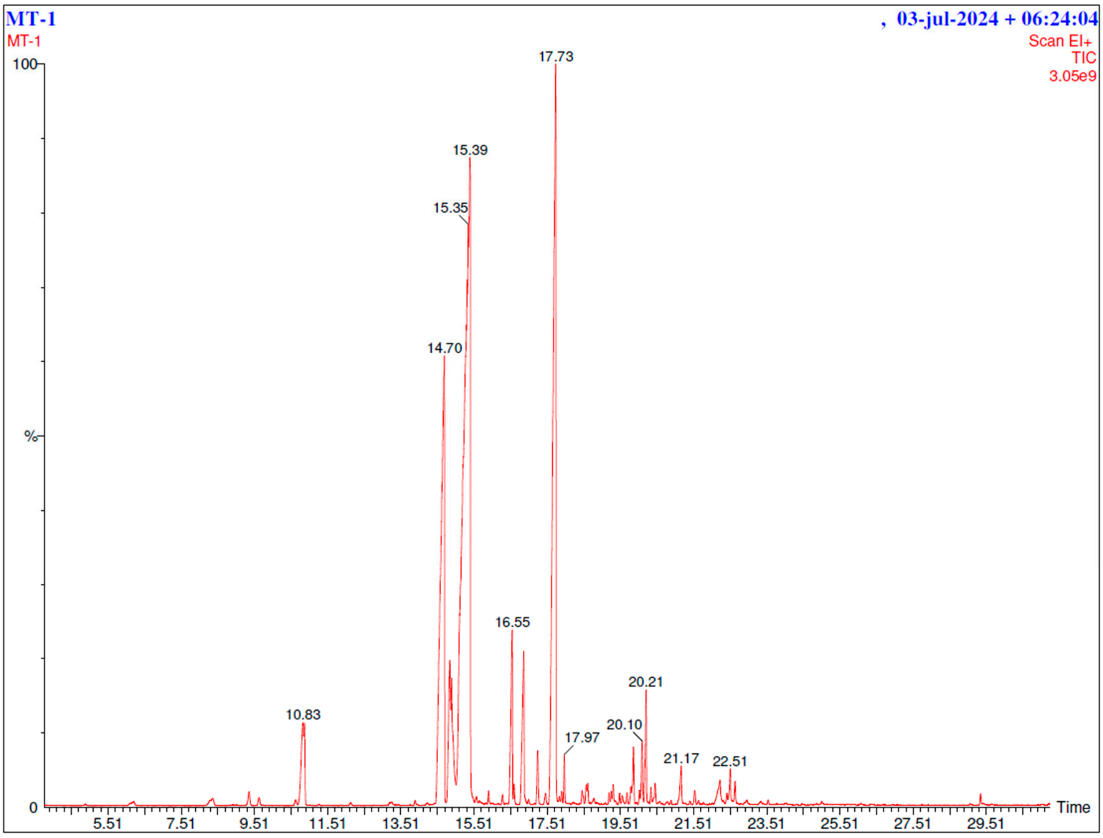


**Figure S3**. Chromatogram obtained by GC-MS of essential oils extracted by steam drag from fresh *M. piperita* leaves.

**Table S1.** Lethal concentration ($LC_{50}$) values of positive control insecticide against *S. frugiperda* larvae, estimated by Probit analysis across larval instars and exposure times.

| Instar | Time (h) | $LC_{50}$ (μg $mL^{-1}$ air) | SE | LCL95 | UCL95 | $\chi^2$ | df | p |
|---|---|---|---|---|---|---|---|---|
| L1 | 24 | 0.159 | 0.021 | 0.123 | 0.206 | 29.89 | 18 | 0.038 |
| | 48 | 0.159 | 0.021 | 0.123 | 0.206 | 29.89 | 18 | 0.038 |
| | 72 | 0.159 | 0.021 | 0.123 | 0.206 | 29.89 | 18 | 0.038 |
| L2 | 24 | 0.190 | 0.023 | 0.150 | 0.240 | 34.54 | 18 | 0.011 |
| | 48 | 0.190 | 0.023 | 0.150 | 0.240 | 34.54 | 18 | 0.011 |
| | 72 | 0.190 | 0.023 | 0.150 | 0.240 | 34.54 | 18 | 0.011 |
| L4 | 24 | 0.248 | 0.025 | 0.203 | 0.303 | 45.53 | 18 | <0.001 |
| | 48 | 0.248 | 0.025 | 0.203 | 0.303 | 45.53 | 18 | <0.001 |
| | 72 | 0.248 | 0.025 | 0.203 | 0.303 | 45.53 | 18 | <0.001 |

**Note**: $LC_{50}$ values were estimated by Probit analysis. SE = standard error; LCL95 and UCL95 = lower and upper 95% confidence limits; $\chi^2$ = chi-square goodness-of-fit statistic; df = degrees of freedom; p = associated probability value.
